# Semiparametric Estimation of Task-Based Dynamic Functional Connectivity on the Population Level

**DOI:** 10.3389/fnins.2019.00583

**Published:** 2019-06-21

**Authors:** Maria A. Kudela, Mario Dzemidzic, Brandon G. Oberlin, Zikai Lin, Joaquín Goñi, David A. Kareken, Jaroslaw Harezlak

**Affiliations:** ^1^Safety and Observational Statistics, Takeda R&D Data Science Institute, Takeda Pharmaceuticals, Cambridge, MA, United States; ^2^Department of Neurology, Indiana University School of Medicine, Indianapolis, IN, United States; ^3^Department of Radiology, Indiana University School of Medicine, Indianapolis, IN, United States; ^4^Department of Psychiatry, Indiana University School of Medicine, Indianapolis, IN, United States; ^5^School of Industrial Engineering, Purdue University, West Lafayette, IN, United States; ^6^Purdue Institute for Integrative Neuroscience, Purdue University, West Lafayette, IN, United States; ^7^Weldon School of Biomedical Engineering, Purdue University, West Lafayette, IN, United States; ^8^Department of Epidemiology and Biostatistics, School of Public Health, Indiana University, Bloomington, IN, United States

**Keywords:** dynamic functional connectivity, semiparametric mixed models, statistical methods, functional MRI, gustatory task, addiction

## Abstract

Dynamic functional connectivity (dFC) estimates time-dependent associations between pairs of brain region time series as typically acquired during functional MRI. dFC changes are most commonly quantified by pairwise correlation coefficients between the time series within a sliding window. Here, we applied a recently developed bootstrap-based technique (Kudela et al., 2017) to robustly estimate subject-level dFC and its confidence intervals in a task-based fMRI study (24 subjects who tasted their most frequently consumed beer and Gatorade as an appetitive control). We then combined information across subjects and scans utilizing semiparametric mixed models to obtain a group-level dFC estimate for each pair of brain regions, flavor, and the difference between flavors. The proposed approach relies on the estimated group-level dFC accounting for complex correlation structures of the fMRI data, multiple repeated observations per subject, experimental design, and subject-specific variability. It also provides condition-specific dFC and confidence intervals for the whole brain at the group level. As a summary dFC metric, we used the proportion of time when the estimated associations were either significantly positive or negative. For both flavors, our fully-data driven approach yielded regional associations that reflected known, biologically meaningful brain organization as shown in prior work, as well as closely resembled resting state networks (RSNs). Specifically, beer flavor-potentiated associations were detected between several reward-related regions, including the right ventral striatum (VST), lateral orbitofrontal cortex, and ventral anterior insular cortex (vAIC). The enhancement of right VST-vAIC association by a taste of beer independently validated the main activation-based finding (Oberlin et al., 2016). Most notably, our novel dFC methodology uncovered numerous associations undetected by the traditional static FC analysis. The data-driven, novel dFC methodology presented here can be used for a wide range of task-based fMRI designs to estimate the dFC at multiple levels—group-, individual-, and task-specific, utilizing a combination of well-established statistical methods.

## Introduction

The assessment of dynamic functional connectivity (dFC), estimated by finding the time-varying association between time series of brain region pairs, is a recent expansion of functional connectivity (FC). Traditional FC analyses, which assume constant functional associations across time (i.e., static metrics) successfully grouped brain regions into distinct functional networks (Greicius et al., 2003; Beckmann et al., 2005; Fox et al., 2005; Yeo et al., 2011). Despite its success, static functional connectivity only partially answers the question of how networks communicate (Turk-Browne, 2013), and it may not detect important network behaviors critical for understanding the brain (Calhoun et al., 2013). The dynamic nature of the functional connectivity in large-scale brain networks during task-free designs (Chang and Glover, 2010) revealed that the brain is never truly at rest. Recent studies pointed out non-stationary nature of functional connectivity that was changing not only during task-related activity but also while resting (Hutchison et al., 2012), and indicated that population-based static networks are less informative to uncover neurological illness (Hutchison et al., 2012; Jones et al., 2012). Similarly, cognitive control processes are transient and dynamic and may be best characterized in terms of inter-regional functional coupling dynamics (Hutchison and Morton, 2016). Others also addressed the dynamic nature of brain activity in both task-based and task-free designs (Debener et al., 2006; Sadaghiani et al., 2009; Doucet et al., 2012; Cribben et al., 2013; Allen et al., 2014), suggesting an important role for dFC analyses in quantifying time-varying network behavior. While dFC methodology was largely developed in healthy subject datasets, it has been employed in clinical populations as well (Filippini et al., 2009), including Alzheimer's disease (Jones et al., 2012), autism (Starck et al., 2013), and schizophrenia (Sakoglu et al., 2010). Too often, however, dFC is estimated solely in a resting state, without any emitted behavior (Turk-Browne, 2013).

A variety of approaches have been developed to assess dynamic functional connectivity, including a sliding window approach (Sakoglu et al., 2010; Jones et al., 2012; Leonardi and Van de Ville, 2013), time-frequency analysis (Chang and Glover, 2010; Yaesoubi et al., 2015), change-point analysis (methods used to detect the important transient point Cribben et al., 2013), data-driven approaches from a signal processing field (Calhoun et al., 2014; Calhoun and Adali, 2016), and dynamic graph methods (Mucha et al., 2010; Fornito et al., 2016). Among the most popular is the sliding window approach due to its simplicity, easy implementation, and ability to recover salient features of dFC. This method has some limitations, such as the window size choice and the inherent variation present in the estimate. Even if there is no association between signals, one can misinterpret their associations as time-varying changes in connectivity (Lindquist et al., 2014). The choice of the window length is an ongoing topic of interest. Previous studies indicated optimal window to be in the 30–60 s range to represent the dynamic nature of the signal (Keilholz et al., 2013; Leonardi and Van de Ville, 2015; Deng et al., 2016; Liégeois et al., 2016), but others suggest using a multilayer formalism indicating importance of both the medium time windows of size 75–100 s and shorter window lengths that reveal individual differences that were not apparent at longer time scales (Telesford et al., 2016).

Many extensions were proposed to overcome the sliding window approach limitations, including tapered windows (Handwerker et al., 2012; Allen et al., 2014; Damaraju et al., 2014) or methods based on multivariate bootstrapping (Kudela et al., 2017). In some cases, the sliding window approach was utilized in combination with methods from the signal processing field, such as higher-order singular value decomposition (Leonardi and Van de Ville, 2013), an independent component analysis (ICA) (Kiviniemi et al., 2011), group ICA (Calhoun and Adali, 2012), or an extension of ICA methods called independent vector analysis (Adali et al., 2014; Ma et al., 2014). Others combined ICA and sliding window methods with k-means clustering method (Allen et al., 2014; Damaraju et al., 2014). However, fMRI data are noisy, which limits signal processing family of methods when they attempt to combine subject-level ICA results. It is not guaranteed that for each subject, the components will be unmixed alike (Calhoun and Adali, 2012; Preti et al., 2017).

Yet another class of methods uses graph theory and applies network analysis to obtain time courses of graph measures, which allows an assessment of dynamically changing associations from a different perspective. The one of most popular graph theory metrics is modularity, which quantifies partition into modules containing brain regions with intra-connectivity greater than obtained by chance (Fornito et al., 2016; Contreras et al., 2019). Mucha et al. (2010) proposed an approach to examine modularity dynamically across time, which was later utilized to investigate dynamic associations during task performance (Bassett et al., 2011, 2015). For more detailed reviews of dFC methods please refer to Hutchison et al. (2013), Calhoun et al. (2014), Preti et al. (2017).

The statistical methodology described in this work was developed to uncover and characterize the dynamics of brain networks during task-based functional magnetic resonance imaging (fMRI) studies. Specifically, we wanted to establish the feasibility of estimating time-varying FC and its confidence intervals in a task-based fMRI. We implemented the method using a novel two-step estimation approach. First, we applied a bootstrap-based approach (Kudela et al., 2017) that utilized a multivariate linear process bootstrap (Jentsch and Politis, 2015) and a sliding window technique to obtain the time-varying functional associations among brain regions in each subject. Then, these subject-specific dFC estimates were treated as an outcome in the semiparametric additive mixed model (Ruppert et al., 2003) to estimate stimulus-specific population-level dFC for each pair of brain regions. This approach estimates the complex correlation structure while simultaneously combining information across subjects to yield population level estimates of time-varying associations and their confidence intervals for each stimulus type and their difference. Unlike other methods, this data-driven approach models multiple repeated observations per subject, the experimental structure, subject-level variability, and importantly, provides condition-specific dFC and confidence intervals for the whole brain at the group level.

Here, we applied our novel methodology to fMRI task data in healthy drinkers. A sample of social-to-heavy alcohol drinkers completed an fMRI task that delivered beer and Gatorade® flavors to subjects' mouths. The flavor of beer is a potent conditioned reward stimulus for alcohol intoxication, provoking dopaminergic activity in the brain's reward system even without alcohol (Oberlin et al., 2013, 2015). Therefore, our objective was to estimate time-varying FC and its confidence intervals for each flavor and their difference, using population-level inference in the whole brain. Based on our previous findings of corticostriatal and dopaminergic responses to beer flavor (Oberlin et al., 2016), we hypothesized that functional connectivity within visual, attentional, and somatosensory “upstream” networks would be similar for both flavors. Reasoning that a conditioned reward signal would integrate into executive and introspective monitoring systems, we hypothesized that beer flavor would enhance associations between limbic (Uddin, 2015), frontoparietal (Etzel et al., 2015; Cole et al., 2016), and default mode networks (Whitfield-Gabrieli and Ford, 2012; Bolt et al., 2017)—systems implicated in attentional gating of executive function between resting and active time periods during salient tasks. Oberlin et al. (2016) demonstrated that wanting and desire to drink correlated with the right ventral striatum and medial orbitofrontal activation to beer flavor stimulation during fMRI. Therefore, we also hypothesized that beer, as compared to Gatorade, would enhance functional connectivity within the reward circuitry (striatal and orbitofrontal regions) involved in reward valuation and alcohol cue salience-gating regions of the anterior insular cortex. To validate our method and extend previous findings, we focused on *a priori* regions of interest (ROIs) implicated in alcohol cue responses from Oberlin et al. (2016). More details about selected *a priori* regions and networks can be found in section Brain Networks and Regions of Interest. Our proposed statistical method aimed to assess the dynamics of these alcohol cue responses in the brain's reward circuits by providing dFC metrics and associated confidence intervals not available using traditional functional connectivity approaches.

The article is organized as follows: study design, data acquisition and preprocessing steps, brain networks and regions of interest, as well as statistical framework for proposed methodology are introduced in the Methods section; results are summarized in the Results section, and the Discussion section provides conclusions and a discussion.

## Methods

### Subjects

Task-based analyses of the blood oxygenation level dependent (BOLD) response to beer and Gatorade flavors for these subjects were previously reported in Oberlin et al. (2016). Subjects (Table 1) were recruited from the local community and prior to participation signed an informed consent approved by the Indiana University Institutional Review Board. All were male, right-handed, and in good self-reported physical and mental health, with recent drinking ranging from social-to-heavy. Each reported beer as one of their two most-often consumed alcoholic beverages (see Oberlin et al., 2016 for complete inclusion and exclusion criteria).

**Table 1:** Subject characteristics.

	**Mean ± (SD)**	**Range**	***N* (%)**
Age	24 (2.3)	21–28	
Caucasian	–	–	24 (100%)
Education	15.8 (1.4)	12–19	
% with at least one first degree relative w/AUD			7 (29%)
Drinks per week^a^	14.9 (9.9)	2–33	
Drinks per drinking day^a^	4.9 (3.0)	1–10	
Heavy drinking days per week^a^, ^b^	1.6 (1.4)	0–6	
AUDIT^c^	10.2 (6.3)	3–26	

a *Drinking data are from the Timeline Followback Interview (Sobell et al., 1986)*.

b *4+ or 5+ drinks per day for females and males, respectively (Gunzerath et al., 2004)*.

c *Alcohol Use Disorders Identification Test (Saunders et al., 1993)*.

*SD, Standard Deviation*.

### Experimental Design

**Figure 1:**
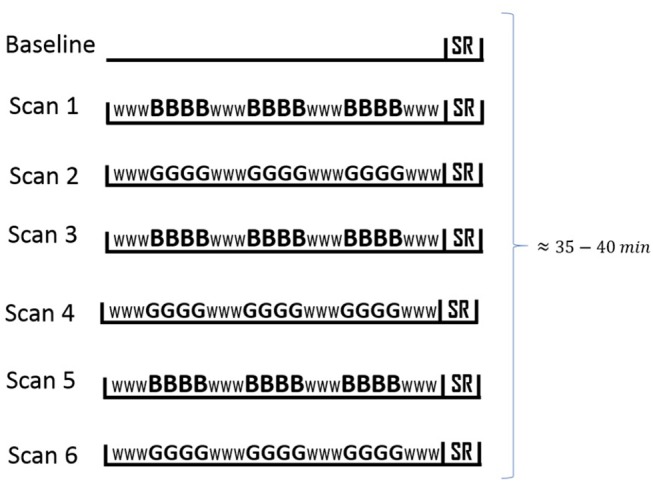
fMRI session outline. Each scan was 4:48 min long, and functional imaging, including subjective ratings typically lasted 35–40 min. Gustatory stimuli (beer, Gatorade©) or water were presented every 11 s during each scan; individual trials are indicated by *B* (beer), *G* (Gatorade©), and *W* (water). The design of separate beer and Gatorade scans was the best match to our earlier PET studies (Oberlin et al., 2013, 2015). SR denotes subjective ratings (pleasantness, intensity, etc., see Oberlin et al., 2016 for details). The scan order is counterbalanced across subjects (beer or Gatorade scan first).

Beer and Gatorade flavors (chosen for their matched flavor intensity; see Oberlin et al., 2013, 2015) were delivered in ~1 s sprays (trials) on subjects' tongues, and were interspersed with neutral water (flavorless sensory baseline). Subjects completed six fMRI scans, with beer and Gatorade scans alternating and flavor order counterbalanced across subjects. In each scan, 3 flavor epochs (4 trials each) were interspersed with 4 water epochs (3 trials each), resulting in 12 flavor and 12 water trials with a fixed 11 s inter-trial interval (Oberlin et al., 2016). Figure 1 illustrates the experimental design scheme.

### Image Acquisition and Pre-processing

Imaging employed a Siemens 3T Magnetom Trio-Tim (Erlangen, Germany) scanner and a 12-channel head coil array. BOLD contrast sensitive functional data (echo planar imaging, gradient echo, 125 volumes, repetition/echo time 2,250/29 ms, flip angle 78°, field of view 220 ×220 mm, 39 interleaved 3-mm thick axial slices, 2.5 ×2.5 ×3.0 mm^3^ voxels, GRAPPA acceleration factor 2) were positioned on a high resolution anatomic volume acquired with a 3D magnetization prepared rapid gradient echo (MPRAGE) sequence. Head movement and motion-related artifacts were minimized using deformable foam pads and real-time three-dimensional prospective acquisition correction (Thesen et al., 2000). Pre-processing was performed with an FSL-based pipeline within Matlab detailed in Contreras et al. (2017) and Amico et al. (2017). Denoised (Coupe et al., 2008; Coupé et al., 2010) T1-weighted MPRAGE volumes were non-linearly transformed (FSL's FLIRT & FNIRT) to the Montreal Neurological Institute (MNI) brain template, which also segmented brain tissues. The inverse transformation then allowed parcellation of the cortical (Shen et al., 2013) and subcortical (Patenaude et al., 2011) gray matter in native BOLD data space. BOLD data preprocessing included slice timing correction, motion correction, registration to T1, detrending, band pass filtering (0.009–0.08 Hz), and normalization to mode 1,000 (Smith et al., 2013).

### FC Data Analysis

FC correlations are especially sensitive to head motion, so we used rigid-body derived realignment parameters and additional metrics to scrub outlier BOLD volumes (Power et al., 2012, 2014) as described in Amico et al. (2017). Five of the initial 29 subjects were excluded because one or more (of the six) BOLD scans contained an excessive fraction (>40%) of BOLD volume outliers, resulting in a final sample of 24 subjects. Physiologic noise and residual head motion confounds were regressed out of signals in eroded masks of the whole-brain gray matter, white matter, and cerebrospinal fluid of the third ventricle, with global signal regression also applied. We employed 3 PCA components from segmented masks to better account for noise (Chai et al., 2012; Power et al., 2015). For each subject, we implemented gray matter parcellation into 278 ROIs, as defined by a meta-analysis of resting state fMRI data (Shen et al., 2013). The time series of each ROI was generated by averaging time series of all voxels within that region.

### Brain Networks and Regions of Interest

Cortical ROIs defined based on Shen et al. (2013) parcellation were assigned to one of the seven resting state networks (RSNs) derived from a large study (*n* = 1,000) of young healthy volunteers (Yeo et al., 2011). Thirty-two non-cortical brain regions were assigned to a subcortical network, while 30 cerebellar regions were discarded due to incomplete BOLD acquisition coverage, yielding 248 cerebral regions used in subsequent analyses.

Many of the brain networks are relevant to the task; either during the cue presentation (primary and associative visual), while following task instructions (attentional), during oral liquid flavor stimulation periods (somatosensory), or in the post-stimulation periods (limbic, frontoparietal, and default mode). The visual and attentional systems should be largely flavor agnostic, while somatosensory regions do exhibit some flavor-specific enhancements in response to Gatorade, which while matched in flavor intensity (see Oberlin et al., 2015, 2016), cannot be perfectly matched to beer in all possible sensory qualities. The prominence of these three “upstream” networks is a prerequisite to studying limbic, cognitive control, and default mode network region responses to alcohol-cue related gustatory stimuli.

While network approaches are commonly used in functional connectivity studies and provide extensive normative data (especially from large resting state studies), it is also of value to understand functional connectivity in the context of specific behavioral circumstances. In the present case, we focused on data derived from a subset of *a priori* regions of interest (ROI) from a study by Oberlin et al. (2016) in which subjects who varied in alcohol drinking behaviors tasted two flavors of different appetitive significance: those of beer and Gatorade. Here we identified Shen et al. regions that included peak activation coordinates of brain areas that: (1) responded to the flavors of beer and Gatorade and, (2) showed differential flavor responses to assign appropriate Shen regions. Each flavor recruited a large network of sensorimotor regions, gustatory cortex (area “G” in the insula/opercular areas), amygdala, and caudate nucleus. We hypothesized that reward regions such as ventral striatum (VST) and orbitofrontal cortex (OFC) would exhibit beer flavor-enhanced associations (Table 2, Oberlin et al., 2016). Of note, the originally reported beer and Gatorade flavor effects (Supplementary Tables S1 and S2 in Oberlin et al., 2016) were relative to the water baseline as is customary in activation studies, while dFC metrics incorporate water trials and are more analogous to the implicit baseline (i.e., resting brain) comparison.

**Table 2:** Models estimating dFC for the two flavors and their difference.

	**Beer f_w=B_(t)**	**Gatorade f_w=G_(t)**	**Subject-specific g_iw_**
dFCM	$\beta_{0}+\beta_{1}t+\sum_{k=1}^{K}u_{k}z_{k}(t)$	$\beta_{0}+\gamma_{0}+(\beta_{1}+\gamma_{1})t+\sum_{k=1}^{K}u_{k}z_{k}(t)+\sum_{k=1}^{K}w_{k}z_{k}(t)$	*b*_*i*0_+*a*_*is*0_
sFCM	*β*_0_	*β*_0_+*γ*_0_	*b*_*i*0_+*a*_*is*0_

### Statistical Methods

Group-level dFC estimates across time for both flavors and their difference for all pairwise ROI associations were obtained in two steps. First, we estimated pairwise ROI associations at a subject level using the method proposed by Kudela et al. (2017). Second, we combined the subject-specific estimates using generalized additive mixed models (Ruppert et al., 2003; Durbán et al., 2005) to obtain a population-level dynamically changing association for each flavor and each pair of regions. In sections Subject-Level dFC Estimation and Population-Level dFC Estimation we provide more details for both steps.

#### Subject-Level dFC Estimation

Whole-cerebrum pairwise dFC associations for each scan were estimated at the subject level by applying a recently proposed technique (Kudela et al., 2017) that combines the sliding time window correlation estimation with an extension of the Multivariate Linear Process Bootstrap (MLPB). The latter is a specialized bootstrap method applicable to bootstrapping time series data (Jentsch and Politis, 2015). Specifically, for each subject and pair of regions, we divided the time-series into adjacent time blocks and used the data within each block to generate bootstrap samples of the bivariate time series via MLPB. We then combined these samples across adjacent blocks to create a bootstrap realization of the whole bivariate time series and estimated pairwise correlations via a sliding window approach with a window size equal to 20 TRs, thus reducing the number of time points from 125 to 105. The correlation coefficient values are between −1 and 1. We applied the Fisher Z-transformation to the estimated correlation coefficients to ensure that the homoskedasticity assumption would not be violated in the GAM approach. The bootstrap procedure was repeated 250 times and the median was used to estimate dFC for each pair of regions and each scan.

In a recent work by Kudela et al. (2017), it was shown that the described bootstrapping procedure provides not only valid model-free time-varying connectivity estimates but also their uncertainty level. In that study, the dFC estimation accuracy was assessed through a series of simulation studies and it was demonstrated that the dFC estimates obtained using the bootstrapping algorithm had a smaller mean squared error than the sliding window technique see Figures 3–7 in Kudela et al. (2017).

Two tuning parameters must be specified for the subject-level dFC estimation: the size of the sliding window and the width of the adjustment block used in the bootstrap procedure. Published empirical results were used as a guideline. One of the key parameters in the sliding window technique is the size of the window, with its' optimization a widely discussed research topic. Shorter windows provide more sensitivity to detect dynamic associations, while longer windows offer a better stratification into regions that provide a core structure, but not necessarily the one of importance. In the literature, suggested optimal window size for the sliding window technique are typically between 30 and 60 s (Sakoglu et al., 2010; Leonardi and Van de Ville, 2015; Telesford et al., 2016). Here, due to the attentional shifts (switching between the resting and active time periods), we decided to use 20 time point window, which translates to the window size of 45 s that should better capture the dynamic nature of the signal. Similarly, the width of adjustment blocks for bootstrapping was selected to be 20 time points. In the future work, the effect of the different window sizes should be examined.

#### Population-Level dFC Estimation

**Figure 2:**
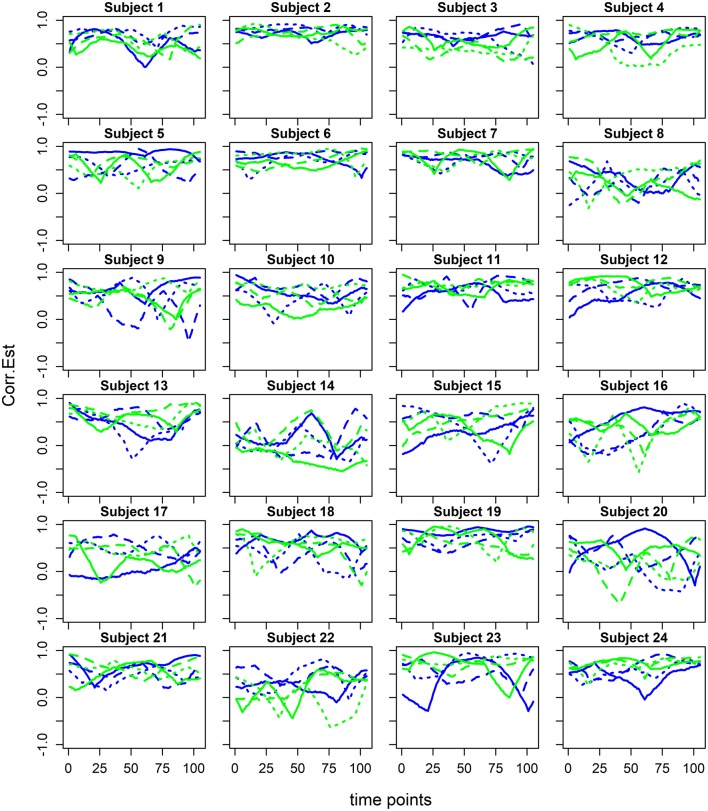
Estimated dynamic functional connectivity associations between the homologous precentral gyri ROIs 34 and 154 from Shen et al. (2013) for beer (blue) and Gatorade (green) scans for each of the study subjects. dFC estimates for each of three beer and Gatorade scans are illustrated by different line styles (solid, dashed, dotted).

Subject-specific dFC estimates were combined using the generalized additive mixed model (GAMM) framework (Ruppert et al., 2003), which is a principled statistical approach accounting for the hierarchical structure of the data and an unspecified (smooth) form of the dFC. GAMM's flexibility permits the estimation of both overall flavor-FC and scan-by-subject-FC. Figure 2 depicts estimated dFC examples for homologous sensorimotor regions (known to activate in this task) for each subject-scan combination. Our specific estimation method relies on a penalized regression approach (see e.g., Ruppert et al., 2003) that accounts for multiple repeated observations per subject while modeling the fixed factors in the experimental design, i.e., three alternating beer and Gatorade scans. This method is also amenable to fast computation in large datasets and facilitated by R's (www.r-project.org) range of mixed models. In our study, we estimated dFC for 30,628 [derived from (n^*^(n+1)/2)-n pairs; where *n* = 248 regions] pairwise associations for each flavor. The details of the statistical model are presented below.

We model dFC as: *dFC*_*ijd*_ = *f*_*d*_(*t*_*j*_)+*g*_*id*_+ε_*ijd*_, where a response variable *dFC*_*ijd*_ is the estimated dynamic FC for each subject *i* (*i* = 1, ⋯ , 24); time point *j* denoted by *t*_*j*_ = 1, ⋯ , 105; and flavor *d* (*d* = *B or G*; *for beer or Gatorade, respectively*). We assume that the error terms, ε_*ijd*_, are independent and normally distributed $N\left(0,\;\sigma_{\varepsilon }^{2}\right)$; *f*_*d*_(*t*_*j*_) is the group-average curve representing the shape of dFC for the flavor *d*; *g*_*id*_ is the subject-specific deviation from the group-average curve for flavor *d*. Based on the experimental design, we assumed that *g*_*id*_ is a sum of two mutually independent random effects: the subject-specific intercept, *b*_*i*0_ and a nested within-subject random scan effect, *a*_*is*0_ where ${\;b}_{i0}\sim \;N\left(0,\;\sigma_{b_{0}}^{2}\right)$, ${\;a}_{is0}\sim \;N\left(0,\;\sigma_{a_{0}}^{2}\right)$, and s (= 1,2,3) denotes a scan number.

The GAMM method enables us to also study the static connectivity (sFCM in Table 2) by assuming that the flavor-specific population-level connectivity is constant, but unknown, i.e., *f*_*B*_(*t*) = β_0_ for beer and *f*_*G*_(*t*) = β_0_ + γ_0_ for Gatorade. As a byproduct of this approach, we obtain the difference between flavors as *c*(*t*) = *f*_*G*_(*t*) − *f*_*B*_(*t*) = γ_0_.

In the dynamic connectivity model (dFCM in Table 2), we assumed that the dFC varied smoothly across time as the brain connectivity varies slowly. The explicit model specification and parameter estimation is presented below. The outcome variable, *y*(*t*), is modeled as a linear combination of basis functions *z*_*k*_(*t*) as $y\left(t\right)=f\left(t\right)+\varepsilon =\;\beta_{0}+\beta_{1}t+\overset{K}{\underset{k=1}{\sum }}u_{k}z_{k}\left(t\right)+\varepsilon$, where *z*_*k*_(*t*) are the O'Sullivan spline basis functions (Wand and Ormerod, 2008), the random effects *u*_*k*_ are independent and follow normal distribution $N\left(0,\;\sigma_{u}^{2}\right)$ and random errors ε are independent and follow a normal distribution $N\left(0,\;\sigma_{\varepsilon }^{2}\;\right)$.

The parameters are estimated by minimizing the criterion $\min\sum_{i=1}^{n}{\left(y_{i}-f\left(t_{i}\right)\right)}^{2}+\lambda \sum_{k=1}^{K}u_{k}^{2}$ (Ruppert et al., 2003). By using the equivalence between the penalized splines estimator of the model and the best linear unbiased estimator of mixed models (Brumback et al., 1999; Durbán et al., 2005), model regression parameters and the smoothing parameter $\lambda =\frac{\sigma_{\varepsilon }^{2}}{\sigma_{u}^{2}}$ are estimated by the restricted maximum likelihood estimation (REML) (see e.g., Ruppert et al., 2003).

The population-level-dFC can then be expressed as: (1) $f_{B}\left(t\right)=\;\beta_{0}+\beta_{1}t+\overset{K}{\underset{k=1}{\sum }}u_{k}z_{k}\left(t\right)$ (for beer), (2) $f_{G}\left(t\right)=\beta_{0}+\gamma_{0}+\left(\beta_{1}+\gamma_{1}\right)t+\overset{K}{\underset{k=1}{\sum }}u_{k}z_{k}\left(t\right)+\overset{K}{\underset{k=1}{\sum }}w_{k}z_{k}\left(t\right)$ for Gatorade. With this flavor-specific function representation, the difference between the flavors can be expressed as $c\left(t\right)=f_{G}\left(t\right)-f_{B}\left(t\right)=\gamma_{0}+\gamma_{1}t+\overset{K}{\underset{k=1}{\sum }}w_{k}z_{k}\left(t\right)$. Parameters *β*_0_, *β*_1_, *γ*_0_, *γ*_1_ are fixed and *u*_*k*_, *w*_*k*_ are random following the normal distributions $N\left(0,\;\sigma_{u}^{2}\right)$ and $N\left(0,\;\sigma_{w}^{2}\right)$, respectively. In our analysis, we used O'Sullivan penalized splines (Wand and Ormerod, 2008) due to their appealing properties including smoothness, numerical stability, natural boundary properties, and a direct generalization of smoothing splines. We define a set of knots κ_1_, …, κ_*K*_ according to quantiles across the time domain and set the number of knots *K* to 40, to allow the dFC function to change non-linearly. The main advantage of penalized splines is that due to a penalty imposed on spline basis coefficients, it is less sensitive to the choice of location and the number of knots (Ruppert et al., 2003; Harezlak et al., 2018). If there are too many knots (risk of overfitting), the unnecessary coefficients will be shrunk toward zero. dFCM and sFCM models are summarized in Table 2.

The steps described in sections Subject-Level dFC Estimation and Population-Level dFC Estimation can be summarized as follows:

- Subject-level dFC estimation:
- a) For each subject and pair of regions, apply the Multivariate Linear Process Bootstrap, a specialized method for bootstrapping time series data (Jentsch and Politis, 2015) to generate bivariate time series bootstrap samples.
- b) Use obtained bootstrap realization of time series for a given pair of regions and apply a sliding window technique to get a set of time-varying estimates of association for each pair of brain regions.
- c) Apply Fisher Z-transformation to the entire set of the bootstrapped dFC trajectories.
- d) Estimate the subject-level dFC for each pair of regions by the median of the dFC trajectories obtained in step 1c.
- Population-level dFC estimation:
- a) Treat subject-specific dFC estimates obtained in Step 1 as repeated measurements within subjects.
- b) Apply penalized splines within the generalized additive mixed model framework to obtain population-level estimates of dFC for both flavors, their difference, and their confidence intervals.

By applying presented algorithm to the gustatory fMRI data, we obtained 30,628 estimates and confidence intervals for flavor specific pairwise dFC curves, including dFC curves for the set of *a priori* defined regions. Finally, we needed to summarize the results in a meaningful and principled way. We detail the dFC summary procedure in sections dFC Summary Measure, Multiple Comparison Correction, and Significance Testing Criteria.

In addition, we performed supplementary analysis for a set of selected *a priori* regions. Here, instead of using bootstrap-based approach for subject-level dFC estimation (step 1 of presented algorithm), we utilized the regular sliding window approach only and compared the results with proposed bootstrap based method (see Supplementary Materials for details).

### dFC Summary Measure

To identify dFC associations of interest, we implemented an objective metric that quantifies the proportion of time during which the confidence intervals around the dFC curve exclude zero on either the positive or negative side. This metric gives a more comprehensive, whole-brain view of the results. For example, 0.8 non-zero coverage indicates that the full confidence interval for the dFC is either above or below zero for 80% of the scan time.

### Multiple Comparison Correction

In our study, we calculated population-level pairwise correlations between 248 brain regions, yielding 30,628 estimates and confidence intervals for flavor specific curves and differences between flavors. To account for multiple comparisons, we applied a false discovery rate (FDR) correction (Benjamini and Yekutieli, 2001) with a 0.05 threshold. We also compared our results with a more stringent family-wise error (FWE) rate correction (Bretz et al., 2010) with the significance level set to 0.05 (see Supplementary Materials).

### Significance Testing Criteria

Informed by the study design, where flavor and water trials in each scan are equally represented, we tested for the proportion of time that the confidence intervals around the dFC curve for beer and Gatorade excluded zero. On average, we expect the proportion of time = 0.5, and ideally, we would only test this during the flavor periods, however temporal smoothing and the sliding window approach precludes such isolated testing. The null hypothesis test for the flavor difference dFC curve was set at a less stringent proportion of time = 0.14, approximating a test for flavor differences during a single flavor block (i.e., 4 flavor trials out of 24 flavor and water trials) adjusted for the sampling limitations of the sliding window approach within the first and last 10 scan time points. In addition, we evaluated significance of flavor differences only when the same pair of regions shows a significant association during the beer scans. To better illustrate the results for a set of *a priori* regions of interest, we present the results using proportion testing at 0.14 and 0.1.

## Results

We present the population-level dFC estimation results in the next section and compare them with the static FC estimates in the following section. The summaries are presented first as dFC curves at the pairwise correlation level, second as all brain parcel pairwise comparisons of the non-zero coverage level, and last as comparisons between the dFCM and sFCM model estimates.

### Dynamic Functional Connectivity Model (dFCM)

**Figure 3:**
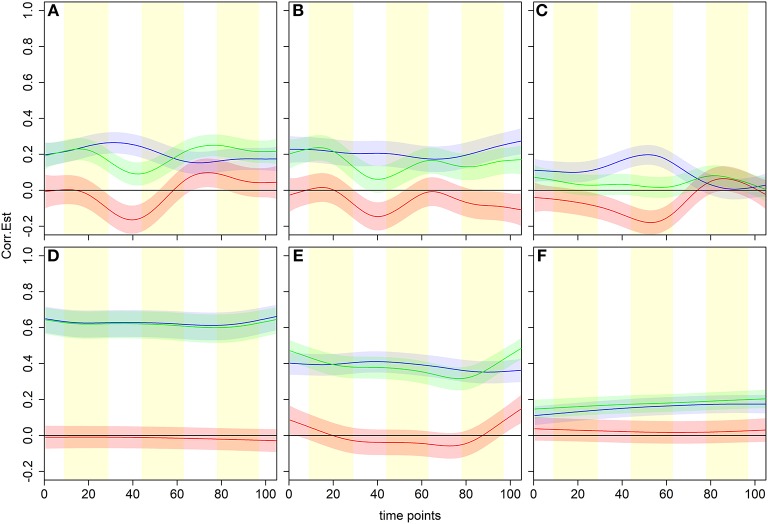
Examples of time dependence for six pairwise associations in the dCFM model. Blue, green and red lines and shaded areas represent estimated dFC with pointwise 95% CIs for beer, Gatorade, and Gatorade-beer difference, respectively, with the vertical yellow shading representing flavor delivery periods. Three scenarios where associations during beer scans are positive and enhanced with respect to Gatorade scans (the difference curve is negative), are shown in the top panels. In all three cases, the difference significantly differs from zero at similar scan times (peaking between time points 40 and 50). The temporal characteristics of the dFC during Gatorade scans, however, differ in amplitude and phase **(A,B)** or have no time points when the associations differ from zero **(C)**. The bottom panels illustrate expected behavior of associations of the right primary sensorimotor cortex (SMC; right precentral gyrus) and three somatomotor network (SMN) regions, indicating no differences between flavors. A homologous, left precentral gyrus area shows an expected, high, nearly constant, positive association for both flavors **(D)**, while a slightly lower positive association is seen to the ipsilateral Rolandic Operculum (RO)/Insula, area “G” of the primary gustatory cortex **(E)**. The ipsilateral Putamen (subcortical part of the SMN) associations are much lower for both flavors, but slowly increase and remain positive **(F)**.

We examined a model of non-linearly changing time-dependent associations between the time series of representative pairs of brain regions (Figure 3). Three scenarios where associations during beer scans are positive and enhanced with respect to Gatorade scans are shown in the top panels. During Gatorade scans, the time dependence of the dFC estimates is either more variable as reflected by estimated amplitude and phase of the curves (Figures 3A,B) or almost constant and not significantly different from zero at any time point (Figure 3C). Other time-varying scenarios for the population-level dFC curve estimates are shown in the remaining panels of Figure 3. To validate dFC estimates, we assessed associations of the right sensorimotor cortex (SMC; precentral gyrus region) to three other somatomotor network (SMN) regions, which showed expected positive associations for both flavors and no significant differences between flavors (Figures 3D–F).

**Figure 4:**
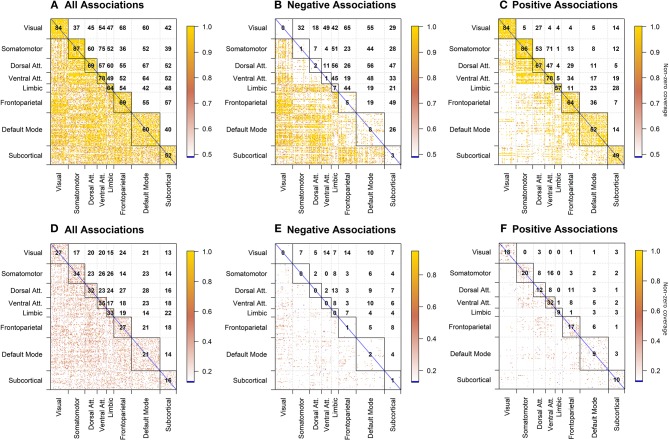
dFC model estimates of all, negative, and positive significant associations for beer (top; **A–C**) and flavor difference (bottom; **D–F**), assessed by testing the proportion of time that the confidence intervals around the dFC curve excluded zero. As the null hypothesis for beer associations, the proportion was set to 0.5 and tested against the alternative that proportion is >0.5. Less stringent proportion value of 0.14 was used for flavor difference testing. All results are corrected for multiple comparisons (*p*_*FDR*_ < 0.05). Each significant association is depicted as a dot in the lower triangular elements while the diagonal and upper triangular elements illustrate a percentage of significant dFC associations between pairs of regions within each network and between networks, respectively.

For clarity, we present the model results in a matrix form, where rows and columns represent pairwise brain region associations. The top panels of Figure 4 illustrate significant associations (left = all, middle = negative only, right = positive only) as derived by the significance testing of the non-zero coverage across time for beer flavor and FDR-adjusted for multiple comparisons within 248 ROIs. The brain regions are organized into 7 cortical resting state networks (RSN; Yeo et al., 2011) and a subcortical set of regions, with the upper triangular and diagonal elements showing the percentage of significant proportions between and within each network, respectively (e.g., 75% of pairwise associations between somatomotor and ventral attention networks are significant). Each colored dot in the lower triangular elements represents the value of the proportion for a specific pair of brain regions. Many of the significant positive associations are observed within the known RSNs, while negative associations tend to occur between regions from different networks (e.g., 56% of time-varying associations between limbic and dorsal attention network regions have a significant negative non-zero coverage; the DMN is more negatively related to other network regions). As anticipated, the difference between flavors summarized in the bottom panels of Figure 4 shows a less regular pattern, reflecting many associations common to both flavors. The highest percentage of significant negative associations (i.e., *f*_*B*_ (*t*) − *f*_*G*_ (*t*) > 0) involves associations of the visual network regions to frontoparietal and ventral attention networks (14%; Figure 4E), while significant positive associations (i.e., *f*_*B*_(*t*) − *f*_*G*_(*t*) <0) are the highest within the ventral attention network (32%; Figure 4F).

### Static Functional Connectivity Model (sFCM)

**Figure 5:**
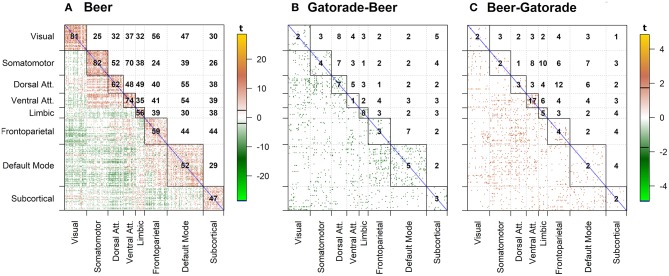
sFCM estimates for β_0_ coefficient representing time-constant associations during beer scans **(A)**. Significant pairwise correlations (pFDR < 0.05; FDR-corrected for multiple comparisons) are shown below the diagonal, while the percentage of significant pairwise ROI associations within- and between- each pair of networks is displayed on and above the diagonal, respectively. None of the sFCM estimates for γ_0_ coefficient representing beer-Gatorade (γ_0_ < 0; **B**) and Gatorade-beer (γ_0_ > 0; **C**) associations satisfy the *p*_*FDR*_ < 0.05 criterion so these effects are presented at *p* < 0.05 (two-tailed, uncorrected for multiple comparisons). The color bars with black horizontal lines indicate *t*-statistic values and appropriate display threshold.

The results presented in Figure 5 are in a matrix form, where each element indicates t-statistic of the model parameters. β_0_ is an estimate of the static (i.e., constant over time) functional connectivity during the beer scans. γ_0_ is an estimate of the static FC for difference between flavors, with negative values indicating associations enhanced by beer (with respect to Gatorade) and positive values indicating associations enhanced by Gatorade (with respect to beer).

#### Beer Associations (**β**_**0**_)

Task-based time-invariant positive associations mirrored established findings from task-free (resting) FC (Yeo et al., 2011) as shown in Figure 5A (*p*_*FDR*_ < 0.05; FDR-corrected for multiple comparisons across all ROIs). Between network interactions were predominantly characterized by positive associations between somatomotor and attention (dorsal and ventral) networks, while frontoparietal-visual, DMN-visual, and DMN-attention (dorsal and ventral) networks showed primarily negative associations.

#### Flavor-Enhanced Associations (**γ**_**0**_)

None of the model estimates for flavor-enhanced associations satisfied the significance criterion (*p*_*FDR*_ < 0.05; FDR-corrected for multiple comparisons). For completeness, the directionality of flavor effects (beer-Gatorade; γ_0_ < 0 and Gatorade-Beer; γ_0_ > 0) is illustrated by Figures 5B,C, respectively presented at an uncorrected significance (*p* < 0.05, two-tailed, single test assumption).

### Model Comparisons of Associations Between *a priori* Regions of Interest

**Figure 6:**
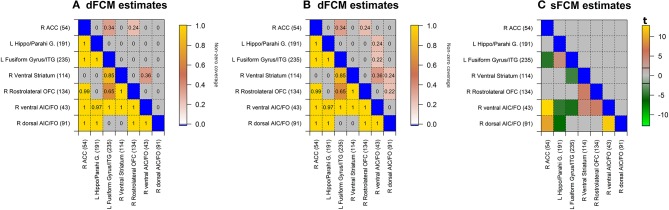
dFCM and sFCM estimates for significant associations between *a priori* regions of interest (for dFCM: **A,B**; for sFCM **C**). Lower triangular elements illustrate associations during the beer scans while upper triangular elements indicate beer-enhanced (i.e., *beer* − *Gatorade*) associations. In the dFC model, non-zero coverage metric for beer was tested for the proportion of 0.5, while the flavor difference was tested for the proportion of 0.14 and 0.1 (**A,B**, respectively). Similarly, **(C)** illustrates sFCM model estimates for β_0_ (beer scan associations; below the diagonal) and γ_0_ coefficient (beer-enhanced associations; above the diagonal). None of the γ_0_ estimates reached the significance criterion *p*_*FDR*_ < 0.05; FDR-adjusted for multiple comparisons in 248 brain regions). The *t*-statistic values displayed in the color bar illustrate the magnitude and direction of observed associations. Matrix elements for which associations do not reach the significance criterion are grayed out. Brain region indices from Shen et al. (2013) are in parentheses. L, left; R, right; md, medial; VST, Ventral Striatum; ACC, Anterior Cingulate Cortex; H & B, Head and Body; vAIC, ventral Anterior Insular Cortex; FO, Frontal Operculum; IFG p.T., Inferior Frontal Gyrus (Pars Triangularis); OFC, Orbitofrontal Cortex; SFG, Superior Frontal Gyrus; MFG, Middle Frontal Gyrus; Hippo/Parahi, Hippocampus/Parahippocampal Gyrus.

To compare the dFCM and sFCM models, we focused on estimated associations between *a priori* ROIs that responded in the general linear model (GLM) “activation” analysis when contrasting [beer > Gatorade] as reported in the Table 2 in (Oberlin et al., 2016). During the beer scans (Figure 6, below the diagonal), the dFCM model detected all associations present in the sFCM model as well as four additional associations. When testing for beer-enhanced associations (Figure 6, above the diagonal), none were found in the sFCM model (Figure 6C). However, the dFCM model detected three and seven significant associations for proportion testing levels of 0.14 and 0.1 (Figures 6A,B respectively).

**Figure 7:**
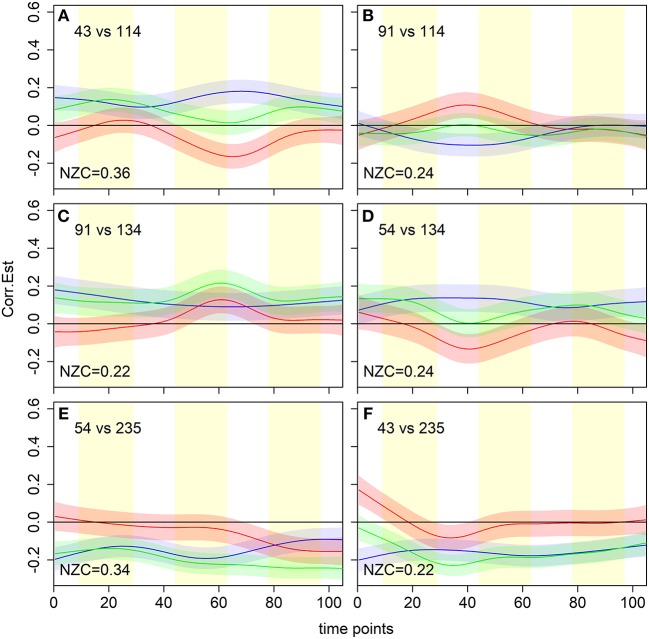
dFCM curve estimates for six (out of seven) significant flavor-enhanced associations between *a priori* regions of interest from Figure 6B. The proportion of time points with non-zero coverage (NZC) is shown in the bottom left of each panel. These results illustrate a variety of scenarios that result in a significant non-zero coverage, with the estimated flavor difference curve either positive **(B,C)**, negative **(A,D,E)**, or both positive and negative **(F)**. Brain region indices from Shen et al. (2013) are: 43 = R-vAIC/FO, 54 = R-ACC, 91 = R-dAIC/Insula, 114 = R-VST, 134 = R-rostrolateral OFC, 235 = L-Fusiform Gyrus. L, left; R, right; VST, Ventral Striatum; ACC, Anterior Cingulate Cortex; vAIC/dAIC, ventral/dorsal Anterior Insular Cortex; FO, Frontal Operculum; OFC, Orbitofrontal Cortex; Hippo/Parahi, Hippocampus/Parahippocampal Gyrus.

Aside from the overall non-zero coverage metric, the dFC model proposed here can be used to further tease apart the time dependence of regional associations as illustrated by Figure 7. For example, non-zero coverage can be broken into sub-classes, depending on whether the dFC estimates are only positive, only negative, or have both positive and negative contributions. In addition, we can select specific cases and evaluate the flavor difference dFC curve only when the beer associations are positive, and the resulting beer-enhanced associations are significant (i.e., estimated flavor difference curve is negative), as in panels A and D. In the second set of scenarios, estimated flavor difference curve is significantly positive (panels B, C), but that occurs due to a negative association during beer scans in one case (panel B), and positive association during Gatorade in another (panel C). Panels E and F still show significant flavor difference associations but in both cases associations during beer and Gatorade scans are negative.

In summary, for each flavor both models yielded regional associations closely resembling resting state networks. However, only the dFC model detected beer flavor-potentiated associations between several reward-related regions, including right ventral striatum (VST), lateral orbitofrontal cortex, and ventral anterior insular cortex (vAIC).

## Discussion

The statistical methodology developed in this work provides a novel approach for the analysis of task-based dynamic functional connectivity. Our approach examines the population–level dFC for a large number of associations between pairs of time-varying processes with a complex correlation structure. We first estimated the time-varying association among 248 functionally defined cerebral regions (Shen et al., 2013) at a subject level using the recently developed algorithm (Kudela et al., 2017). Kudela et al. (2017) showed that the proposed bootstrapping approach provided a valid model-free, time-varying connectivity estimates together with associated confidence bands. The obtained estimates had a smaller MSE compared to the regular sliding-window approach. As a result, we obtained dFC curves and theirs confidence intervals for all 30,628 pairwise time-varying associations for each subject and scan, which included a set of *a priori* regions of interest. Then, these subject-specific estimates were used to obtain a population-level dynamically changing association for each stimulus and their differences via the semiparametric additive mixed effects model approach. This approach allowed us to incorporate the study design by taking into account scan effects as well as subject- and task-specific variability. As a result, we combined the information across subjects and scans to obtain a population estimate of time-varying association and its confidence intervals for each flavor and the difference between flavors.

We also compared the performance of two popular procedures for multiple testing; FDR- and FWER- based correction. The FDR correction for strongly correlated fMRI data was slightly less conservative than the Bonferroni correction, but the resulting number of significant associations was very similar (see Supplementary Materials).

We proposed a novel metric to summarize the results from population-level pairwise associations for each flavor and difference between flavors by using a non-zero coverage of confidence bands and the magnitude/sign of dFC curves. We showed that the dynamic functional connectivity analysis of the gustatory task fMRI data yielded a pattern closely resembling the resting state networks (the networks found when subjects do not perform any task). Recent studies by Cole et al. (2014, 2016) reported that the FC architectures across a variety of tasks were highly similar (80% shared variance) to the resting-state FC architecture. This correspondence also strengthens the validity of the outcome. Presented dFC approach also reproduced known static FC between homologous areas and revealed differences in dFC patterns that standard approaches would likely miss. The non-zero coverage for the difference revealed beer and Gatorade differentiation in visual, somatomotor, and attentional networks, as well as frontoparietal and default mode networks. Intercepts from the static FC model, interpreted as the mean dFC across time for each flavor individually, also yielded FC patterns similar to the RSNs. However, no associations for the difference between flavors were significant after adjusting for multiple comparisons in the static model. It should be noted that our approach is fully data-driven and uses the resting state networks only to relate the estimated task-based dFC to the known brain organization.

As a validation, we performed analysis for a set of *a priori* regions of interest, which were previously demonstrated to be involved in alcohol cue response (Oberlin et al., 2016). A more targeted analysis showed that ventral striatal, lateral orbitofrontal, and insular regions had time courses that were all positively associated most during beer scans (and more than during Gatorade), confirming their specificity to the response to an alcohol paired cue. The methods used in the (Oberlin et al., 2016) study were GLM-based and implemented in SPM (www.fil.ion.ucl.ac.uk/spm/). While this co-activation approach has been in use for a long time, it is hypothesis driven and assumes identical canonical hemodynamic response (HDR) in all brain areas and subjects. The chemosensory aspect of the gustatory stimuli (taste of beer and Gatorade), however, is not optimally described using a rigid HDR framework. The possibility of sustained responses in some reward-related areas as well as different temporal dependence of BOLD responses in sensory-related and salience-assigning regions (e.g., insula) is better assessed by applying a data-driven approach such as implemented in this work. Indeed, our dFC analysis showed patterns and time dependence of associations that might not be detectable with standard approaches.

One of the limitations of the proposed approach is that temporal smoothing introduced by the sliding window application precludes a disentangling of flavor and water stimulus contributions. In other words, reported flavor results might be diminished by the presence of water trials. One solution would be a finer temporal sampling (e.g., subsecond repetition time with multiband acquisition), which would increase the number of measurements 2–3 times. Nevertheless, we were able to reproduce Beer>Water and Gatorade>Water contrast results from Oberlin et al. (2016). Another limitation is that our approach considers only associations between two brain regions at a time and further extension is needed for more than two regions. To fully benefit from the large sample of estimated dFC curves, an application of clustering algorithms would allow us to investigate different dFC curve classifications. Consequently, one could uncover and test specific time dependence scenarios not easily modeled in the standard FC or GLM-based approaches. In future studies, we plan to extend our dFC analysis to these other domains, as well as to replicate the regions showing specificity to beer flavor. The results presented here were also obtained using a modest size data sample. Hence, more detailed interpretation of presented associations should be left for the follow-up investigations, with the present manuscript speaking to the feasibility of the method.

We also compared the performance of the algorithm for a set of *a priori* regions of interests using the regular sliding window approach only during the subject-level estimation of dFC (step one of the proposed algorithm; please see Supplementary Materials for details). In general, two approaches yielded similar results. The shape and behavior of dFC trajectories were generally similar, but the bootstrap-based approach provided smoother estimates. Proposed method and the regular sliding window only approach presented similar temporal dependence between pairs of brain regions but differed in detecting the number of statistically significance associations. Nevertheless, the advantages of each approach can only be fully assessed with simulation studies using a realistic data generation mechanism. Further simulation studies with a realistic data generation mechanism would have to be undertaken to conclusively show superior performance. Similar studies have been performed in the Kudela et al. (2017) manuscript under numerous scenarios.

In summary, we demonstrated the importance and utility of the proposed methodology when modeling population-level dFC that can be implemented with the statistical significance criteria applicable at different spatial resolutions—from the whole-brain, to the network level, or even a subset of *a priori* regions. The proposed approach is data-driven and provides flexible methodology to investigate associations between brain regions' time series. More specific to the brain's reward system in those who drink alcohol, the approach showed enhancement of the right ventral striatum and ventral insular cortex association by beer, independently validating the main activation-based finding from Oberlin et al. (2016), and providing a novel insight into the dynamics of beer-potentiated regional associations.

## Ethics Statement

This study was carried out in accordance with the recommendations of The Indiana University Institutional Review Board (IRB) IRB00000219 in compliance with 21 C.F.R. 56.109 (e) and 46 C.F.R. 46.109 (d) with written informed consent from all subjects. All subjects gave written informed consent in accordance with the Declaration of Helsinki. The protocol was approved by The Indiana University Institutional Review Board (IRB) IRB00000219.

## Author Contributions

MK and JH developed and implemented the statistical models and their application to functional imaging data. DK, BGO, and MD contributed conception and design of the imaging study. MK performed data analyses and wrote the first draft of the manuscript, with feedback provided by JH and MD. ZL helped with additional computations and analysis. JH, MD, DK, BGO, and JG wrote sections of the manuscript. All authors contributed to manuscript revision, read, and approved the submitted version.

### Conflict of Interest Statement

MK was employed by company Takeda Pharmaceuticals. The remaining authors declare that the research was conducted in the absence of any commercial or financial relationships that could be construed as a potential conflict of interest.
