# Supplementary material for: Semiparametric Estimation of Task-Based Dynamic Functional Connectivity on the Population Level
Source: Front Neurosci. 2019 Jun 21;13:583. doi: 10.3389/fnins.2019.00583 (PMC6598619; doi:10.3389/fnins.2019.00583)
Supplement: Supplementary file 1 [file Data_Sheet_1.docx]

Supplementary Material

Semiparametric estimation of task-based dynamic functional connectivity on the population level

**Maria A Kudela^1^, Mario Dzemidzic^2,4^, Brandon G Oberlin^2,3^, Zikai Lin^3^, Joaquín Goñi^5,6,7^, David A Kareken^2,3,4^, Jaroslaw Harezlak^8*^**

*** Correspondence:** Jaroslaw Harezlak, harezlak@iu.edu

# Multiple comparison correction

In this study, we estimated pairwise associations between 248 brain regions and tested their significance accounting for multiple comparisons. We applied a commonly used false discovery rate (FDR) correction developed by Benjamini & Yekutieli (2001) and compared the results using another popular, more stringent family-wise error (FWE) rate correction (Bretz et al. 2010). The significance criterion for both methods was set at corrected p < 0.05. The number of significant associations as a function of the proportion value for both methods is presented in Supplementary Figures 1 and 2. As expected, both multiple comparison adjustment methods showed number of significant association decreasing with the increasing proportion level. The FDR correction for strongly correlated fMRI data was slightly less conservative than the Bonferroni correction, but the resulting number of significant associations was very similar.


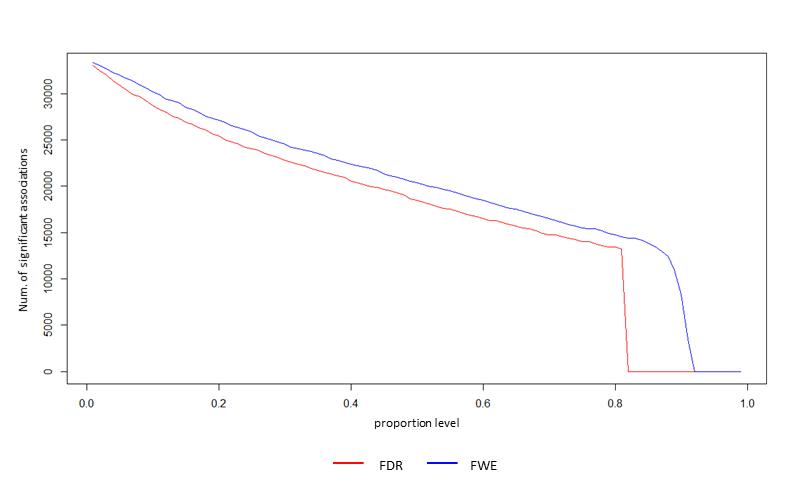


**Supplementary Figure** 1. Number of significant associations for beer flavor versus a level of proportion for FDR and FWE corrections (red and blue line, respectively).


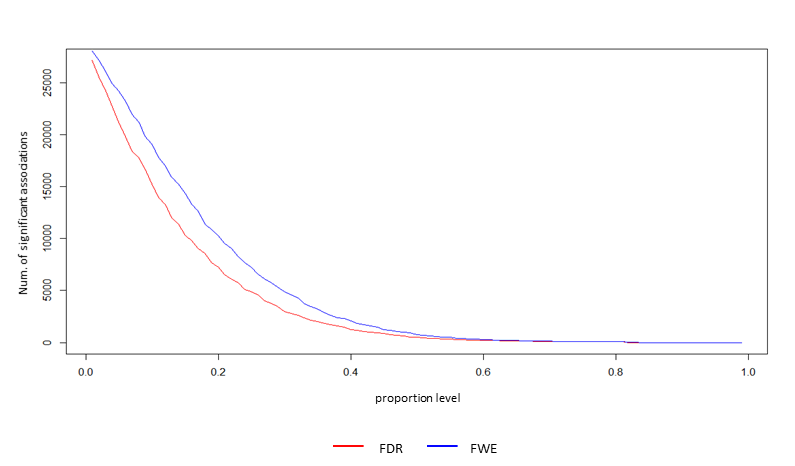


**Supplementary Figure** 2. Number of significant associations for the flavor difference between beer and Gatorade as a function of the proportion level for FDR and FWE corrections (red and blue line, respectively).

# Comparison of the population level dFC estimate results when using bootstrap-based and the regular sliding window approach for subject-level dFC estimation

We also compared population-level results for a set of *a priori* regions of interest (as presented in Figure 6) when subject-specific dFC estimates in step one of the proposed algorithm were obtained using a bootstrap-based algorithm and when applying the regular sliding window approach only (RSWO). The window length was identical in both approaches. In general, two methods yielded very similar results. Proposed bootstrap-based approach detected significant associations for 16 out of 21 pairwise comparisons during the beer scans, while the RSWO approach detected one less significant association (see lower triangular elements of Figure 6 A and Supp. Figure 3 A, respectively). For the flavor difference and proportion testing of 0.14 (presented in this manuscript), proposed method detected three significant beer-enhanced associations (upper triangular elements of Figure 6 A). The RSWO detected four additional significant associations between regions (upper triangular elements of Supp. Figure 3 A). For the flavor difference and proportion testing of 0.1, the proposed algorithm detected seven significant differences, while RSWO approach detected ten (upper triangular elements of Figure 6 B and Supp. Figure 3 B, respectively). The shapes of dFC trajectories were generally similar, but the proposed method provided smoother estimates (Supp. Figure 4) than the RSWO approach. For both approaches temporal dependence of pairwise associations was generally similar, but the defined significance testing criteria differed.

In summary, we applied both the bootstrapping and RSWO approach for step one in our proposed approach. The advantages of each approach can be fully assessed only with simulation studies using a realistic fMRI data generation mechanism. Nevertheless, bootstrap-based estimator was previously shown to have better properties than the regular sliding window technique (Kudela et al. 2017), with the latter also demonstrated to show inherent variation present in the estimate, which may lead to erroneous conclusions (Lindquist et al. 2014). Similar comparison studies were performed in the Kudela et al. (2017) manuscript under numerous scenarios.


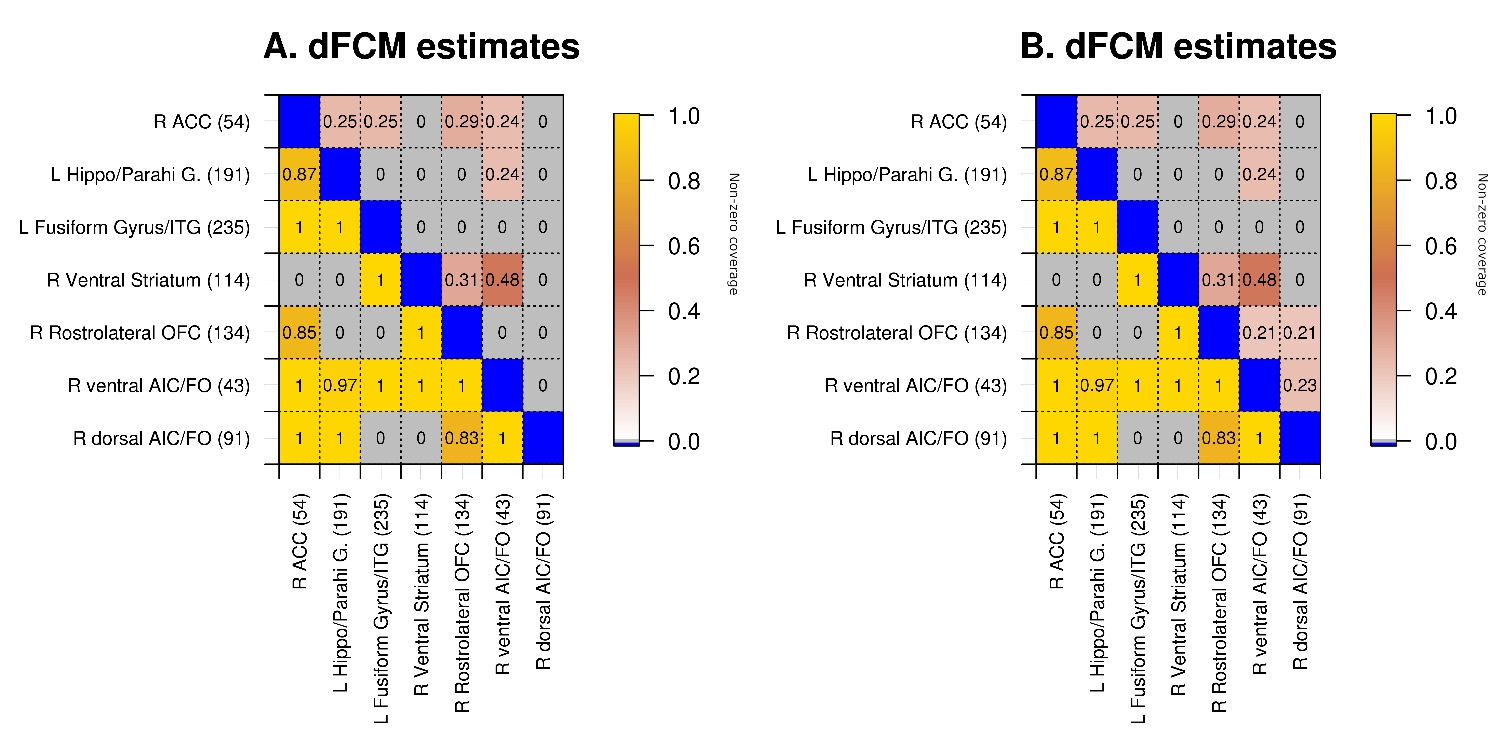


**Supplementary Figure** 3. dFCM estimates for significant associations between *a priori* regions of interest using the sliding window approach only (i.e., without using the bootstrapping step). Similarly as in Figure 6, lower triangular elements illustrate associations during the beer scans while upper triangular elements indicate beer-enhanced (i.e., $beer-Gatorade$) associations. The non-zero coverage metric for beer was tested for the proportion of 0.5 (lower triangular elements of panels A and B, same for both panels), while the flavor difference was tested for the proportion of 0.14 and 0.1 (upper triangular elements of panels A and B, respectively). Brain region indices from Shen et al. 2013 are in parentheses. Abbreviations: L – left, R – right, md – medial, VST – Ventral Striatum, ACC – Anterior Cingulate Cortex, H & B – Head and Body, vAIC – ventral Anterior Insular Cortex, FO – Frontal Operculum, IFG p.T. – Inferior Frontal Gyrus (Pars Triangularis), OFC – Orbitofrontal Cortex, SFG – Superior Frontal Gyrus, MFG – Middle Frontal Gyrus, Hippo/Parahi – Hippocampus/Parahippocampal Gyrus.


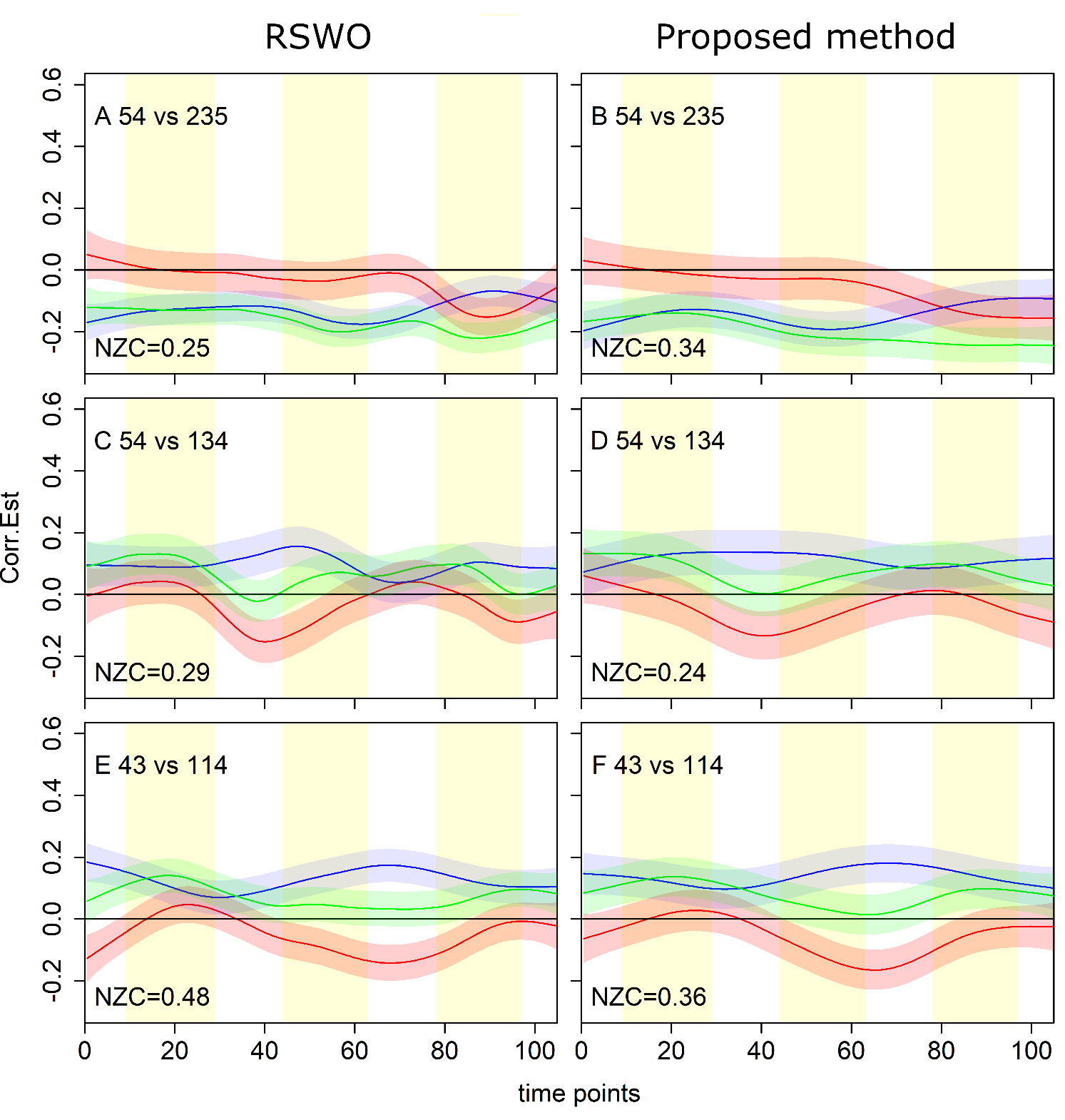


**Supplementary Figure** 4. dFCM curve estimates for three significant beer flavor-enhanced associations between *a priori* regions of interest from Figure 6 A. Left: dFCM curve estimates obtained without bootstrap step. Right: dFCM curve estimates obtained using proposed method. The proportion of time points with non-zero coverage (NZC) is shown in the bottom left of each panel. Brain region indices from Shen et al. 2013 are: 43 = R-vAIC/FO, 54 = R-ACC, 114 = R-VST, 134 = R-rostrolateral OFC, 235 = L-Fusiform Gyrus. L – left, R – right, VST – Ventral Striatum, ACC – Anterior Cingulate Cortex, vAIC – ventral Anterior Insular Cortex, OFC – Orbitofrontal Cortex.
